# Geographic Disparities in Access to Glaucoma Surgery: Lessons from a Nationwide Registry Study

**DOI:** 10.3390/jcm15114357

**Published:** 2026-06-04

**Authors:** Jeppe Nygård Samuelsen, Christina Eckmann-Hansen, Jens Rovelt, Hadi Kjærbo, Kim Holmgaard, Miriam Kolko

**Affiliations:** 1 Department of Drug Design and Pharmacology, University of Copenhagen, 2100 Copenhagen, Denmark; 2 Scandinavian Eye Center, 2900 Hellerup, Denmark; 3 Dine Øjenlæger, 2960 Rungsted Kyst, Denmark; 4 Department of Ophthalmology, Rigshospitalet, 2600 Glostrup, Denmark; 5 Department of Ophthalmology, Aalborg University Hospital, 9000 Aalborg, Denmark

**Keywords:** glaucoma, glaucoma surgery, registry data

## Abstract

**Background**: Glaucoma is a progressive, age-related optic neuropathy and a leading cause of irreversible blindness worldwide. Ensuring timely diagnosis and equitable access to surgical care is therefore essential to prevent avoidable vision loss. **Methods**: Nationwide registry-based data on hospital-based glaucoma diagnoses and surgical procedures over an 11-year period were analyzed and stratified by treatment region and area of residence. Population data were age-stratified to allow calculation of standardized diagnosis and surgery rates per 10,000 population aged ≥ 60 years. Regional comparisons were performed, and demographic projections for populations aged ≥ 60 years were generated using an exponential smoothing state space model. **Results**: Geographic variation in glaucoma care was observed, despite broadly similar demographic profiles across regions. Surgery-to-diagnosis ratios among individuals aged ≥ 60 years differed markedly between regions. Relevant for future healthcare planning, age forecasting suggests an increase in people aged ≥ 60 years over the coming years. **Conclusions**: Geographic disparities in glaucoma surgical care may persist even in well-resourced healthcare systems. Centralization of surgical services may contribute to differences, although explanations such as variation in referral patterns and clinical decision-making cannot be excluded. These findings highlight a broader, internationally relevant challenge: aligning glaucoma care delivery with shifting demographic needs.

## 1. Introduction

**Figure 1:**
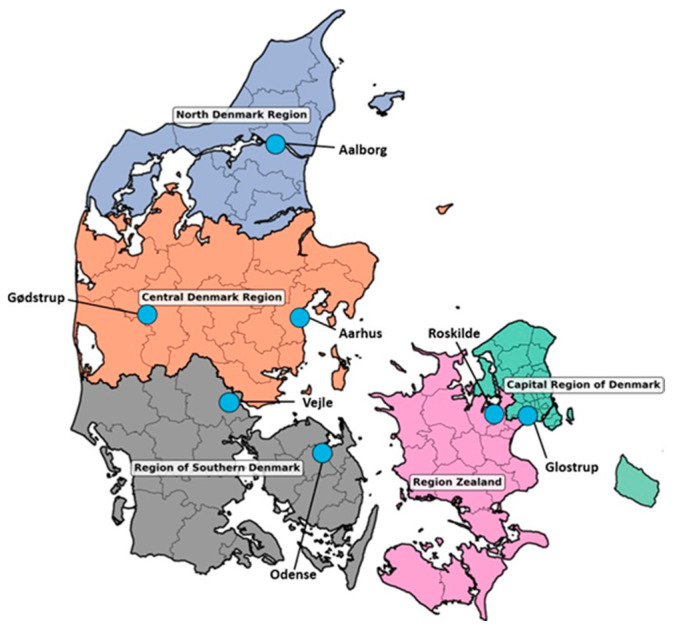
Map of Denmark visualizing the division of regions and municipalities. The locations for glaucoma surgery are shown as blue dots.

Healthcare systems in many high-income countries are organized into regional structures responsible for delivering hospital-based care to defined populations. Denmark represents one such model, currently comprising five administrative regions and 98 municipalities (see Figure 1). Within this system, patients are generally referred and treated within their own region unless highly specialized care is required.

Glaucoma surgery in Denmark is highly centralized and only performed within the public hospitals, with procedures performed at a limited number of locations (7 out of 29 public hospitals), serving large geographic catchment areas [1]. This configuration reflects a broader international trend toward centralization of specialized surgical services. However, such models may introduce access challenges, particularly for patients living in rural or underserved areas, where travel distances are longer and the density of referring ophthalmologists is lower [2,3]. These structural factors are not unique to Denmark but are observed across many healthcare systems where service consolidation has been implemented.

At the same time, demographic changes are reshaping the demand for glaucoma care globally. Life expectancy continues to increase, and the proportion of elderly individuals is rising across most European countries and other high-income settings [4]. In Denmark, glaucoma prevalence exceeds 6% by the age of 70 and increases steeply thereafter [5,6]. Consistent with international patterns, more than 80% of glaucoma surgeries are performed in individuals aged 60 years and above [7]. As populations age, the demand for timely surgical intervention is therefore expected to grow substantially [8,9,10].

Despite universal healthcare coverage, the interaction between centralized surgical capacity, regional demographic variation, and the geographic distribution of ophthalmic services may contribute to unequal access to care [1,3]. Similar challenges have been described internationally, where factors such as travel distance, referral pathways, and local resource availability influence not only disease detection but also the likelihood of receiving timely surgical treatment and appropriate follow-up [2].

Using Denmark as a model system, this study investigates whether measurable regional- and municipality-level differences exist in glaucoma diagnosis and surgical treatment. We hypothesized that glaucoma surgical activity would vary substantially across Danish regions and municipalities, reflecting differences in healthcare organization, demographic composition, and geographic access to specialized ophthalmic services. We further hypothesized that projected population ageing may increase future regional pressure on glaucoma-related healthcare services. These findings are intended to provide insights that are applicable beyond Denmark, highlighting structural and demographic factors that may contribute to variation in glaucoma treatment in other healthcare systems facing similar organizational and demographic transitions. While previous studies have examined disparities in glaucoma care utilization and geographic access to ophthalmic services, few have evaluated nationwide variation in glaucoma surgical activity within a universal healthcare system.

## 2. Materials and Methods

### 2.1. Data Collection

Count data from Danish hospitals were collected based on 10 ICD-10 diagnosis codes and 40 procedure codes of glaucoma surgeries from the period 2013 to 2023 on the regional and municipality levels. Regional-level data were defined as the region in which the treatment was carried out for the patient. Municipality-level data were defined as the municipality of residence for the patient. Glaucoma diagnoses and surgeries from the period 2013 to 2023 were grouped on the 5 Danish regions and on the 98 Danish municipalities. The data were analyzed as aggregated counts of recorded procedures and hospital diagnosis registrations. The data were collected from the Danish National Patient Register (LPR) [11] (via https://www.esundhed.dk/Emner/Operationer-og-diagnoser/Landspatientregisteret-Avanceret-udtraek, accessed 10 December 2024). All diagnoses defined as glaucoma were included. See Appendix A. To determine glaucoma-related codes we used the European Glaucoma Society Guidelines for Glaucoma [12]. For surgeries, all surgeries related to glaucoma according to the Danish Health Data Authority were included. Non-invasive surgery types such as selective laser trabeculoplasty and iridotomy were excluded, as these types of non-invasive laser surgeries are also performed in certain ophthalmic private practices and not only in hospitals. An overview of procedure codes included in the study can be found in Appendix B. Diagnosis and surgery data points (at a specific year and municipality/region) with less than five counts were suppressed and set to zero prior to data access due to national data protection regulations. Therefore, values below 5 were indistinguishable from true zero values in the dataset. Population data grouped by age from the total Danish population was collected from Statistics Denmark from the time period 2013–2024 (via https://www.statistikbanken.dk/20021, accessed 10 December 2024). A yearly mean of four quarterly population counts from the register FOLK1A was calculated for each year for the whole country and for each region. For municipalities, the count of citizens grouped per one year of age was collected from the register BY2. These data were also grouped on the 5 Danish treatment regions and on the 98 Danish municipalities of residence, allowing population- and age-adjusted glaucoma analyses.

### 2.2. Statistical Analysis

Statistical analyses were performed with Python version 3.12 using Cursor and R version 4.5.1 using RStudio.

### 2.3. Calculation of Normalized Surgery and Diagnosis Rates

For each region *r* ∈ {1, 2, …, 5} and municipality *m* ∈ {1, 2, …, 98} at year *t*, normalized surgery and diagnosis rates were calculated per 10,000 population aged 60 years and above.

#### 2.3.1. Surgery Rate

Given that the risk of glaucoma increases with age and that people aged 60 years and above account for the majority of glaucoma cases, an age-adjusted population denominator was used to calculate surgery rates. The surgery rate for region *r* (or municipality *m*) at year *t* was calculated as

(1) $$Surgery\;Rate\;=\frac{S_{r,t}}{P_{60+,r,t}}\times 10,000$$

where *S_r_*_,*t*_ denotes the total number of surgery procedures in region *r* (or municipality *m*) during year *t*, and *P*_60+,*r*,*t*_ represents the total population aged 60 years and above in region *r* (or municipality *m*) at year *t*.

A descriptive, hypothesis-generating comparison of surgery rates between Western Denmark and Eastern Denmark was applied. Here, Western Denmark was defined as the North Denmark Region, the Central Denmark Region, and the Region of Southern Denmark. Eastern Denmark was defined as the Capital Region of Denmark and Region Zealand. For this comparison, the municipality of residence numbers were used. The groups were normalized based on the average total population of Western Denmark and Eastern Denmark, respectively, in the study period.

#### 2.3.2. Diagnosis Rate

Hospital diagnosis rates were also calculated using an age-adjusted population denominator. The diagnosis rate for region *r* (or municipality *m*) at year *t* was calculated as

(2) $$Diagnosis\;Rate=\frac{D_{r,t}}{P_{60+,r,t}}\times 10,000$$

where *D_r_*_,*t*_ denotes the total number of glaucoma diagnoses in region *r* (or municipality *m*) during year *t*, and *P*_60+,*r*,*t*_ represents the total population aged 60 years and above in region *r* (or municipality *m*) at year *t*.

### 2.4. Negative Binomial Regression

To analyze regional trends of surgery rates across the five Danish regions (2013–2023), a negative binomial regression [13] model with region-specific time trends was employed. A negative binomial model was chosen as preliminary inspection of the data showed strong overdispersion, with the variance exceeding the mean of the surgery counts. Model fit was evaluated by comparing observed and predicted counts and through inspection of residual patterns, which did not indicate major lack of fit.

For each region, a negative binomial regression model with the following specification was fitted:

log(*µ_it_*) = *β*_0_ + *β*_1_Years*_t_*+ log(Pop_60+,*it*_) (3)

where *µ_it_* is the expected surgery count for region *i* in year *t*, Years*_t_* is years since 2013 (0–10), and Pop60+,*it* is an offset term to adjust for regional differences in the population aged 60 years and above. The negative binomial regression analysis was designed as a descriptive ecological assessment of regional trends in glaucoma-related surgical activity rather than inferential patient-level models. Accordingly, the findings based on this analysis should be interpreted as population-level associations, and causal or individual-level inferences cannot be drawn from the aggregated registry data.

### 2.5. Surgery-to-Diagnosis Ratio

To assess treatment intensity, the ratio of glaucoma surgeries to diagnoses (surgery-to-diagnosis ratio, %) for each region and municipality over the 2013–2023 period was calculated.

This metric represents the relation between the count of aggregated hospital glaucoma diagnoses and the count of aggregated glaucoma surgical procedures. Municipalities with unavailable diagnosis counts during the study period were excluded from the analysis, as surgery-to-diagnosis ratios could not be meaningfully calculated for these locations. Surgery-to-diagnosis ratios were calculated as

(4) $$Surgery-to-diagnosis\;Ratio=\frac{S_{r,t}}{D_{r,t}}\times 100\%$$

where *S_r_*_,*t*_ denotes the total number of surgeries in region *r* (or municipality *m*) during year *t*, and *D_r_*_,*t*_ denotes the total number of hospital diagnoses in region *r* (or municipality *m*) during year *t*.

### 2.6. Age Forecasting Model

Using the historical population data of age distribution in Denmark, an additive error, damped additive trend, no seasonal, exponential smoothing state space (AAdNETS) forecast model was applied to predict the development in the proportion of people aged 60 years and above until 2030 [14]. We chose ETS modelling for the forecast over more complex approaches such as cohort-component projection which requires detailed birth, death, and migration data unavailable for our study and ARIMA (AutoRegressive Integrated Moving Average) models, which require longer time series. ETS provides a transparent, well-established method suitable for short-term demographic forecasts with limited historical data. The forecast model was applied across five municipality groupings specified in Section 2.7 after aggregating counts.

### 2.7. Definition of Urban and Rural Areas

The grouping of urban and rural areas was defined on a municipality level as per the official definitions by Statistics Denmark: https://www.dst.dk/da/Statistik/temaer/land-og-by (accessed 10 December 2024). The classifications are based on city size and job accessibility: Hovedstadskommuner (Capital municipalities: ≥200,000 accessible jobs), Storbykommuner (Metropolitan municipalities: cities with ≥100,000 inhabitants and <200,000 accessible jobs), Provinsbykommuner (Mid-sized city municipalities: cities with ≥30,000 inhabitants and <200,000 accessible jobs), Oplandskommuner (Commuter municipalities: <30,000 inhabitants and ≥40,000 accessible jobs), and Landkommuner (Rural municipalities: <30,000 inhabitants and <40,000 accessible jobs).

## 3. Results

### 3.1. Surgery Rates

**Figure 2:**
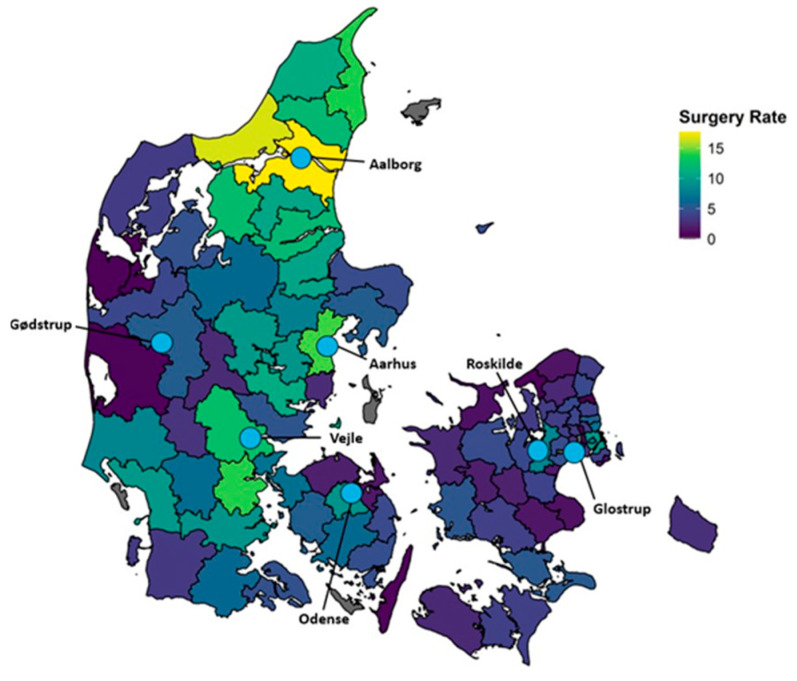
Surgery rates in Danish municipalities, 2013–2023 average. Surgery rates were calculated as counts of surgery procedures per 10,000 population aged 60 years and above. Darker colours indicate low surgery rates, while lighter colours indicate high surgery rates. Municipalities with zero registered surgeries in the period are shown as grey.

Average surgery rates for municipalities during the period 2013–2023 are shown in Figure 2. Clear geographical differences were observed across the country. Overall, surgery rates were higher among residents of municipalities in Western Denmark than Eastern Denmark, with particularly high rates in municipalities within the North Denmark Region. For the full overview of municipality surgery rates, see Appendix C.

#### Western vs. Eastern Denmark

**Figure 3:**
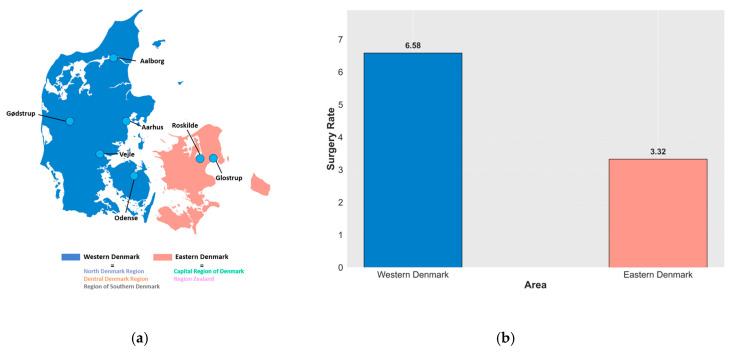
Descriptive comparison of surgery rates between Western and Eastern Denmark. (**a**) Geographical representation of Western and Eastern Denmark. (**b**) Surgery rates for Western Denmark and Eastern Denmark, 2013–2023 average. Surgery rates were calculated as counts of surgery procedures per 10,000 population aged 60 years and above.

Over the study period, Western Denmark had an average total population of approximately 3.2 million people with an average age of 41.9 years and five glaucoma surgery sites. Eastern Denmark had an average total population of approximately 2.8 million people with an average age of 41.5 years and two glaucoma surgery sites. An overview can be found in Table 1. The average surgery rate for Western Denmark during the study period was 6.58. In Eastern Denmark the average surgery rate during the period was 3.32. See Figure 3.

### 3.2. Negative Binomial Regression Analysis

Negative binomial regression models revealed heterogeneity in trends of glaucoma surgery rates across Denmark’s five regions. An overview of the results of the models can be found in Table 2.

**Figure 4:**
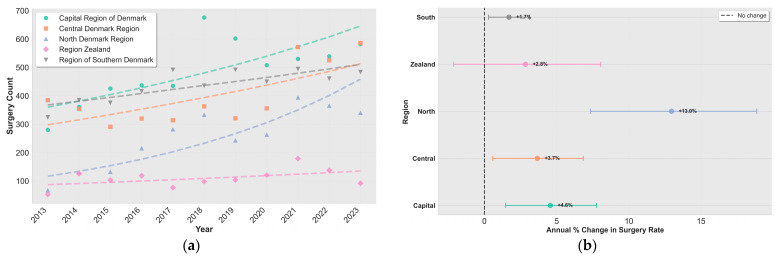
Negative binomial regression analysis of surgery count data for each of the five Danish regions in the period 2013–2023. (**a**) Observed annual glaucoma surgery counts (markers) and negative binomial model predictions (dashed lines) by region, 2013–2023. (**b**) Annual percentage change in surgery rates by region from negative binomial regression model, 2013–2023. Error bars represent 95% confidence intervals.

All five regions demonstrated positive trends, indicating overall increasing surgery rates over the 2013–2023 period in the population aged 60 years and above (Figure 4). However, the magnitude of these increases varied substantially, as did the raw number of surgeries per region (Figure 4). The North Denmark Region exhibited the steepest increase with a rate ratio of 1.130. Region Zealand was the only region that did not show a statistically significant increase. The Region of Southern Denmark exhibited the smallest significant increase, with a rate ratio of 1.017.

The large difference in annual growth rates between the North Denmark Region and the other four regions indicates variation in the utilization of glaucoma surgery. Over the 11-year study period, these differential trends have resulted in greater divergence of regional surgery rates.

### 3.3. Diagnosis Rates

Hospital diagnosis rates were calculated for municipalities in Denmark. Diagnosis rates exhibited geographic variation across Danish municipalities; however, we did not observe clear regional patterns when analyzing diagnosis rates on their own. For the full overview of diagnosis rates, see Appendix D.

### 3.4. Surgery-to-Diagnosis Ratios

**Figure 5:**
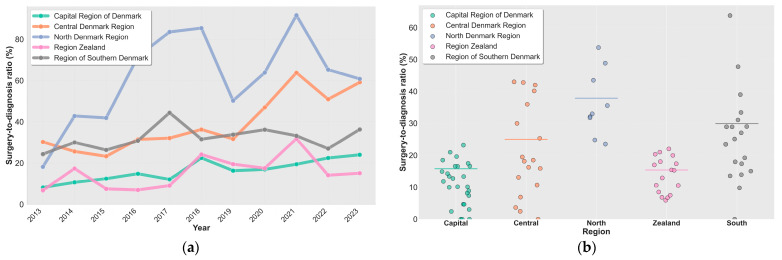
Surgery-to-diagnosis ratios (%) in Danish regions and municipalities for the period 2013–2023. (**a**) Surgery-to-diagnosis ratios (%) in Danish regions for the period 2013–2023. (**b**) Average surgery-to-diagnosis ratios (%) in Danish municipalities grouped by the five regions for the period 2013–2023. Region-wide municipality averages are shown as horizontal lines.

The surgery-to-diagnosis ratios varied substantially across regions, revealing disparities in treatment patterns (Figure 5). Western regions demonstrated higher surgery-to-diagnosis ratios than Eastern regions throughout the study period.

The North Denmark Region demonstrated the highest surgery-to-diagnosis ratios, averaging 61.2% over the study period (range: 18.1–91.7%). The Central Denmark Region showed an increasing trend from 30.2% in 2013 to 59.1% in 2023, with notable acceleration after 2019. The Region of Southern Denmark maintained relatively stable ratios, averaging 31.3%. In contrast, Eastern regions demonstrated substantially lower surgery-to-diagnosis ratios. The Capital Region of Denmark averaged 16.1% while Region Zealand demonstrated the lowest surgery-to-diagnosis ratio, averaging 14.8%.

At the municipality level, ratios among municipalities (n = 90) ranged from 0% to 63.8%. Substantial within-region variation was observed across all regions.

### 3.5. Age Forecasting

**Figure 6:**
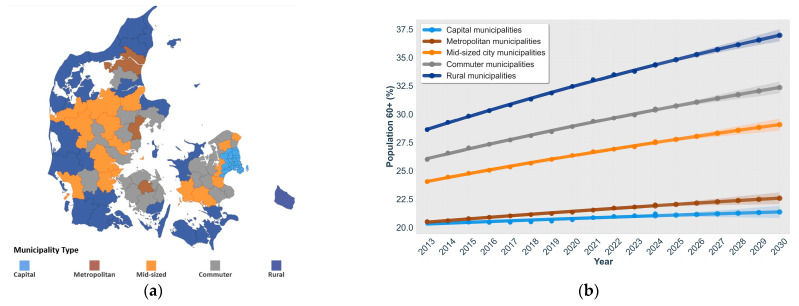
ETS forecast of proportion (%) of people aged ≥ 60 for five municipality types, defined by Statistics Denmark. (**a**) Geographical visualization of the five different types of municipalities as defined by Statistics Denmark. (**b**) ETS forecast of population share of people aged ≥ 60 (%) in the five types of municipalities with 95% confidence intervals.

As described in Section 2.6, an AAdNETS forecast model was used to make a short-term prediction of the increase in the proportion of people aged 60 years and above in Denmark. An overview of the forecasting model results can be found in Table 3. The forecast shows differences in demographic ageing trajectories (Figure 6). By 2030, rural municipalities are projected to reach 36.97% aged 60+, a 2.63 percentage point (pp) increase from 2024. In contrast, capital municipalities show minimal ageing at 21.37%, only a 0.17 pp increase. Intermediate municipality types show a clear urbanization gradient: commuter municipalities 32.35% (+1.89 pp), mid-sized city municipalities 29.07% (+1.50 pp), and metropolitan municipalities 22.58% (+0.59 pp). The urban–rural (capital–rural) disparity reaches 15.60 pp by 2030.

## 4. Discussion

This population-based registry study suggests geographic variation in glaucoma surgical care over more than a decade. While the data are derived from a single national healthcare system, the observed patterns reflect challenges that are highly relevant across many high-income countries. Even within a universal healthcare model, marked differences in surgery rates can persist between regions with otherwise comparable demographic profiles.

### 4.1. Geographic Disparities and Healthcare Infrastructure

The findings suggest that differences in healthcare organization, including the degree of centralization of specialized surgical services, may contribute to the observed geographic variation in hospital-based surgical activity. In this setting, regions with fewer surgical centres relative to their population exhibited lower surgical rates, suggesting that infrastructure density is a potential determinant of treatment availability. Importantly, the present registry-based analyses cannot determine the extent to which these patterns reflect differences in clinical need, disease severity, access to care, or healthcare delivery. The observed geographic variation may reflect differences in healthcare organization, referral pathways, coding practices, or concentration of specialized ophthalmic services. As such, the findings should be interpreted as descriptive evidence of geographic disparity rather than direct evidence of undertreatment, reduced access, or capacity limitations. Similar patterns have been reported internationally, where centralization of subspecialized services, although intended to improve quality and efficiency, may inadvertently create access barriers for geographically dispersed populations [15,16].

Consistent with international evidence, substantial within-region variation was observed. Rural–urban gradients are widely documented across healthcare systems and are associated with delayed diagnosis, reduced treatment uptake, and poorer outcomes in chronic eye diseases [17].

In addition, the concentration of surgical services may influence not only access but also system dynamics such as capacity expansion, innovation, and procedural adoption. Health services research suggests that limited provider density and reduced competition can contribute to lower procedural volumes and slower implementation of new techniques, further widening regional gaps in care [18].

### 4.2. Demographic Change and Future Demand

In many countries, the proportion of individuals aged 60 years and above is rising, with particularly rapid growth in rural and non-metropolitan areas. Given that glaucoma prevalence increases steeply with age, typically affecting 4–10% of individuals over 60, this demographic shift is expected to increase demand for both medical and surgical management [6,19].

While population ageing is not necessarily a direct predictor of surgical volume, it does represent a major and universal driver of increasing glaucoma burden. Future healthcare planning should align with evolving demographic trends to combat potential geographic imbalances [16,20].

### 4.3. Surgery-to-Diagnosis Variability

Variation in the relation between the number of hospital diagnoses and the number of surgical procedures was observed both between and within regions [15,17]. Overall surgical activity appeared low compared to other high-income countries [6]. Disparities in the uptake of newer surgical technologies have been reported internationally and are often linked to reimbursement structures, training opportunities, and organizational factors [21,22].

### 4.4. Implications for Healthcare Systems

Taken together, these findings illustrate a broader, globally relevant challenge: aligning glaucoma care delivery with evolving demographic and geographic realities. Centralized models of care, while beneficial in some respects, must be balanced against the need for accessibility, particularly in ageing and geographically dispersed populations. Potential strategies to address these challenges include the following:

- Expanding surgical capacity through decentralization or shared-care models;
- Increasing the involvement of non-hospital-based ophthalmologists in surgical care;
- Enhancing referral pathways and outreach to underserved areas.

Such approaches may help reduce geographic differences, improve timely access to care, and better match service provision to population needs.

### 4.5. Strengths and Limitations

The strengths of this study include its large-scale, population-based design and long observation period, enabling detailed assessment of geographic variation and temporal trends. The integration of demographic projections further strengthens its relevance for future healthcare planning.

While the nationwide registry framework provided broad population-level coverage, a major limitation of this approach is the inability to assess individual-level clinical factors such as disease severity, treatment indications, and patient preferences, as well as incomplete capture of diagnoses outside hospital settings. As a result, surgery-to-diagnosis ratios should be interpreted as possible indicators of healthcare delivery rather than precise measures of clinical need.

The aggregation of procedure codes may mask variation in surgical practice patterns. The included procedure codes represent a heterogeneous group of glaucoma-related interventions and may have included non-primary glaucoma procedures, potentially introducing some degree of procedural misclassification. Stratified analyses by procedure type were considered but were limited by low counts in subcategories, particularly at the municipality level, which would reduce statistical robustness and increase the impact of data suppression. Further, the suppression of low counts (<5) may disproportionately affect estimates, especially in smaller municipalities where apparent zero counts may include suppressed low counts rather than the true absence of surgery or diagnosis. Thus, the data suppression potentially leads to downward bias in municipality-level estimates.

In Denmark, patients may receive care outside their region of residence if highly specialized treatment is required. Cross-regional patient mobility may influence the observed patterns, and this could lead to under- or overestimation of surgical activity in specific regions.

## 5. Conclusions

This study highlights geographic disparities within glaucoma surgical care, with marked differences both between and within regions. While the data are drawn from Denmark, similar patterns are likely to exist in many healthcare systems where specialized surgical services are centralized, rural populations are growing, or the distribution of ophthalmic expertise is uneven.

The combination of structural limitations and demographic ageing creates a trajectory in which demand may outpace capacity, potentially placing patients at higher risk of delayed intervention. Such disparities underscore the broader challenge of ensuring equitable access to care in any health system, regardless of funding model or national wealth.

Addressing this challenge may require bringing surgical services closer to patients by leveraging all available ophthalmic expertise. Strategies may include decentralizing care, expanding surgical provision outside highly centralized hospital settings, enabling qualified ophthalmologists to contribute to surgical treatment, and improving referral pathways to optimize patient access.

Future research should evaluate patient-level outcomes to determine whether geographic disparities translate into differences in vision preservation, quality of life, and long-term clinical outcomes. Qualitative studies capturing patient and provider perspectives will be critical to designing targeted interventions that improve access and reduce unwarranted variation.

As populations continue to age globally, ensuring sufficient and equitable access to glaucoma surgery is a priority for healthcare systems worldwide. Proactive planning, capacity expansion, and redistribution of resources are likely essential to reduce the societal burden of glaucoma.

## Figures and Tables

**Table 1:** Comparison of demographics between Western and Eastern Denmark.

	Western Denmark	Eastern Denmark
Average population	~3.2 million	~2.8 million
Average age	41.9 years	41.5 years
Glaucoma surgery sites	5 locations	2 locations

**Table 2:** Annual trends in surgery rates by Danish regions using negative binomial regression.

Region	Rate Ratio (95% CI)	*p*-Value
North Denmark Region	1.130 [1.073, 1.189]	<0.001
Capital Region of Denmark	1.046 [1.015, 1.078]	<0.001
Central Denmark Region	1.037 [1.006, 1.068]	0.002
Region Zealand	1.028 [0.979, 1.080]	0.118
Region of Southern Denmark	1.017 [1.003, 1.031]	<0.001

Note: Rate ratios of annual changes in surgery rates estimated using negative binomial regression models. Values greater than 1 indicate increasing trends. *p*-values were evaluated at a 5% significance level.

**Table 3:** Population ageing forecasts by municipality type: 2024 baseline and 2030 projections.

Municipality Type	2024 (%) Aged 60+	2030 (%) Aged 60+	Absolute Change	Relative Change
Rural municipalities	34.34	36.97	+2.63 pp	+7.6%
Commuter municipalities	30.46	32.35	+1.89 pp	+6.2%
Mid-sized city municipalities	27.57	29.07	+1.50 pp	+5.5%
Metropolitan municipalities	21.98	22.58	+0.59 pp	+2.7%
Capital municipalities	21.20	21.37	+0.17 pp	+0.8%

Note: Forecasts based on ETS (A, Ad, N) models with damped trend fitted to 2013–2024 population data. Values for 2030 represent point estimates with 95% confidence intervals found in Figure 6. pp = percentage points.

## Data Availability

The surgery and diagnosis count data presented in this study are available on request from the corresponding author as it is no longer available from the original website (https://www.esundhed.dk/Emner/Operationer-og-diagnoser/Landspatientregisteret-Avanceret-udtraek, accessed 10 December 2024). The population data presented in the study are openly available via https://www.statistikbanken.dk/20021 (accessed 10 December 2024) and the registers FOLK1A and BY2.

## Abbreviations

The following abbreviations are used in this manuscript:

ETS	Error, Trend, Seasonal
AAdNETS	Additive error, Additive Damped trend, No seasonal ETS
ARIMA	Auto-Regressive Integrated Moving Average
pp	Percentage points
NA	Not available

## Appendix A

**Table A1:** ICD-10 glaucoma diagnosis codes included in the study.

ICD-10 Code	Diagnosis
DH400	Glaucoma suspect
DH401	Primary open-angle glaucoma
DH402	Primary angle-closure glaucoma
DH403	Traumatic glaucoma
DH404	Glaucoma secondary to eye inflammation
DH405	Glaucoma secondary to other eye disease
DH406	Drug-induced glaucoma
DH408	Other forms of glaucoma
DH409	Glaucoma, unspecified

## Appendix B

**Table A2:** Glaucoma surgery procedures included in the study.

Procedure Code	Procedure Description	Procedure Group
KCHB00	Laser synechiolysis	Other glaucoma related surgeries
KCHB10	Puncture and irrigation of anterior chamber	Other glaucoma related surgeries
KCHB15	Puncture of anterior chamber with aqueous humor exchange	Other glaucoma related surgeries
KCHB20	Goniotomy	Angle surgery
KCHB30	Goniopuncture	Angle surgery
KCHB40	Goniosynechiolysis	Angle surgery
KCHB50	Trabeculotomy	Filtering surgery
KCHB60	Anterior synechiolysis	Other glaucoma related surgeries
KCHB65	Posterior synechiolysis	Other glaucoma related surgeries
KCHB99	Other operation on anterior chamber or angle	Other glaucoma related surgeries
KCHC10	Peripheral iridectomy	Other glaucoma related surgeries
KCHC20	Sector iridectomy	Other glaucoma related surgeries
KCHC30	Central iridectomy	Other glaucoma related surgeries
KCHD05	Laser dilatation of microchannel	Other glaucoma related surgeries
KCHD10	Trabeculectomy	Filtering surgery
KCHD15	Trabeculectomy with/without iridectomy	Filtering surgery
KCHD20	Trepanation of sclera	Other glaucoma related surgeries
KCHD30	Trabeculectomy with laser	Filtering surgery
KCHD31	Phacotrabeculectomy	Filtering surgery
KCHD35	Trabeculectomy with drainage implantation	Filtering surgery
KCHD36	Revision oftrabeculectomy	Revision
KCHD40	Iridencleisis	Other glaucoma related surgeries
KCHD41	Needling of filtration bleb	Revision
KCHD50	Creation of shunt to anterior chamber	Drainage devices
KCHD50A	Creation of large shuntin anterior chamber	Drainage devices
KCHD50B	Creation of small shuntin anterior chamber	Drainage devices
KCHD60	Deepsclerectomy without implant	Other glaucoma related surgeries
KCHD65	Deepsclerectomy with implant	Other glaucoma related surgeries
KCHD99	Other filtration operation	Filtering surgery
KCHE10	Excision of pathological tissue in iris	Other glaucoma related surgeries
KCHE20	Laser treatment of pathological tissue in iris	Other glaucoma related surgeries
KCHE99	Other operation on pathological tissue in iris	Other glaucoma related surgeries
KCHF00	Excision of pathological tissue in ciliary body	Other glaucoma related surgeries
KCHF05	Transscleral laser or ultrasound coagulation of ciliary body	Cyclodestructive procedures
KCHF10	Transpupillary laser coagulation of ciliary body	Cyclodestructive procedures
KCHF15	Cryotherapy of ciliary body	Cyclodestructive procedures
KCHF20	Electrocoagulation of ciliary body	Cyclodestructive procedures
KCHF30	Cyclodialysis	Cyclodestructive procedures
KCHF99	Other operation on ciliary body	Cyclodestructive procedures
KCHW99	Other operation on anterior chamber, angle, iris or ciliary body	Cyclodestructive procedures

## Appendix C

**Table A3:** Average glaucoma surgery rates by municipality (2013–2023).

Municipality	Region	Surgery Rate
Aalborg	North	17.44
Jammerbugt	North	16.35
Aarhus	Central	14.12
Kolding	South	13.85
Frederikshavn	North	13.75
Vejle	South	12.40
Vesthimmerlands	North	12.20
Mariagerfjord	North	11.93
Brønderslev	North	11.92
Hjørring	North	10.85
Horsens	Central	10.71
Randers	Central	10.26
Rebild	North	10.22
Esbjerg	South	9.68
Silkeborg	Central	9.68
København	Capital	9.39
Haderslev	South	9.36
Odense	South	9.26
Skanderborg	Central	9.18
Roskilde	Zealand	8.17
Varde	South	8.10
Frederiksberg	Capital	7.86
Favrskov	Central	7.71
Gentofte	Capital	6.99
Fredericia	South	6.82
Vejen	South	6.66
Middelfart	South	6.65
Viborg	Central	6.26
Faaborg-Midtfyn	South	6.19
Aabenraa	South	6.18
Lyngby-Taarbæk	Capital	5.77
Herning	Central	5.52
Assens	South	5.52
Syddjurs	Central	5.49
Vordingborg	Zealand	5.27
Slagelse	Zealand	5.19
Greve	Zealand	4.95
Hedensted	Central	4.89
Skive	Central	4.82
Sønderborg	South	4.76
Svendborg	South	4.64
Høje-Taastrup	Capital	4.51
Holbæk	Zealand	4.46
Nyborg	South	4.41
Norddjurs	Central	4.40
Lejre	Zealand	4.32
Fredensborg	Capital	4.31
Holstebro	Central	4.22
Allerød	Capital	4.22
Furesø	Capital	4.18
Køge	Zealand	3.91
Rudersdal	Capital	3.86
Tårnby	Capital	3.80
Næstved	Zealand	3.79
Gladsaxe	Capital	3.74
Morsø	North	3.72
Guldborgsund	Zealand	3.72
Egedal	Capital	3.62
Hvidovre	Capital	3.59
Helsingør	Capital	3.56
Ballerup	Capital	3.53
Frederikssund	Capital	3.47
Thisted	North	3.40
Tønder	South	3.38
Billund	South	3.26
Albertslund	Capital	3.17
Brøndby	Capital	2.85
Bornholm	Capital	2.84
Odder	Central	2.79
Ikast-Brande	Central	2.71
Kalundborg	Zealand	2.55
Lolland	Zealand	2.51
Hillerød	Capital	2.35
Ringsted	Zealand	2.14
Nordfyns	South	2.08
Sorø	Zealand	1.97
Solrød	Zealand	1.80
Faxe	Zealand	1.71
Kerteminde	South	1.61
Gribskov	Capital	1.48
Dragør	Capital	1.39
Odsherred	Zealand	1.37
Stevns	Zealand	1.33
Rødovre	Capital	1.19
Herlev	Capital	0.93
Langeland	South	0.87
Ringkøbing-Skjern	Central	0.78
Struer	Central	0.67
Lemvig	Central	0.66
Halsnæs	Capital	0.65
Hørsholm	Capital	0.52
Fanø	South	0
Glostrup	Capital	0
Ishøj	Capital	0
Læsø	North	0
Samsø	Central	0
Vallensbæk	Capital	0
Ærø	South	0

## Appendix D

**Table A4:** Average glaucoma diagnosis rates by municipality (2013–2023).

Municipality	Region	Diagnosis Rate
Samsø	Central	84.84
Greve	Zealand	46.43
Kolding	South	44.81
Vejle	South	42.76
Gentofte	Capital	42.11
Roskilde	Zealand	41.42
København	Capital	40.70
Frederiksberg	Capital	40.41
Holstebro	Central	39.84
Rudersdal	Capital	39.56
Frederikshavn	North	38.88
Vesthimmerlands	North	38.70
Gladsaxe	Capital	37.53
Skive	Central	36.60
Mariagerfjord	North	36.57
Ballerup	Capital	35.09
Lyngby-Taarbæk	Capital	33.29
Silkeborg	Central	33.20
Brøndby	Capital	32.66
Haderslev	South	32.25
Rebild	North	32.25
Køge	Zealand	30.90
Jammerbugt	North	30.71
Herning	Central	30.59
Herlev	Capital	30.22
Faxe	Zealand	29.06
Hvidovre	Capital	28.14
Lejre	Zealand	28.12
Egedal	Capital	27.89
Odense	South	27.65
Høje-Taastrup	Capital	27.43
Struer	Central	27.34
Middelfart	South	27.29
Faaborg-Midtfyn	South	27.25
Hedensted	Central	27.23
Brønderslev	North	27.21
Rødovre	Capital	27.05
Frederikssund	Capital	26.92
Tårnby	Capital	26.78
Sønderborg	South	26.77
Ringsted	Zealand	26.26
Solrød	Zealand	26.02
Horsens	Central	25.40
Furesø	Capital	25.31
Holbæk	Zealand	25.20
Vordingborg	Zealand	25.10
Næstved	Zealand	25.08
Helsingør	Capital	24.52
Randers	Central	24.17
Vejen	South	23.98
Ishøj	Capital	23.90
Skanderborg	Central	23.78
Slagelse	Zealand	23.73
Fredensborg	Capital	23.70
Norddjurs	Central	23.48
Billund	South	22.87
Nyborg	South	22.72
Hørsholm	Capital	22.56
Hjørring	North	22.13
Favrskov	Central	22.05
Glostrup	Capital	21.91
Syddjurs	Central	21.89
Odsherred	Zealand	20.78
Hillerød	Capital	20.76
Dragør	Capital	19.92
Tønder	South	19.90
Vallensbæk	Capital	19.52
Lemvig	Central	19.16
Sorø	Zealand	19.12
Stevns	Zealand	18.82
Gribskov	Capital	18.80
Guldborgsund	Zealand	18.49
Fredericia	South	17.63
Kerteminde	South	17.47
Odder	Central	17.25
Varde	South	17.05
Ikast-Brande	Central	16.89
Svendborg	South	16.10
Morsø	North	15.90
Halsnæs	Capital	15.44
Esbjerg	South	15.18
Viborg	Central	15.05
Lolland	Zealand	14.91
Kalundborg	Zealand	14.73
Thisted	North	14.15
Nordfyns	South	13.92
Bornholm	Capital	13.73
Ringkøbing-Skjern	Central	10.90
Langeland	South	6.22
Ærø	South	5.16
Aarhus	Central	NA
Albertslund	Capital	NA
Allerød	Capital	NA
Assens	South	NA
Fanø	South	NA
Aalborg	North	NA
Aabenraa	South	NA
Læsø	North	NA
